# Accidental Central Venous Catheter Placement in the Internal Thoracic Vein: A Case Report

**DOI:** 10.7759/cureus.9255

**Published:** 2020-07-18

**Authors:** Patrick Goodin, Nikita Jain, Hafiz Muhammad Jeelani, Anchit Bharat

**Affiliations:** 1 Anesthesiology, Indiana University School of Medicine, Indianapolis, USA; 2 Internal Medicine, Chicago Medical School, Rosalind Franklin University of Medicine and Science, McHenry, USA; 3 Internal Medicine, Rosalind Franklin University of Medicine and Science, McHenry, USA; 4 Internal Medicine, Indiana University Health Ball Memorial Hospital, Muncie, USA

**Keywords:** central venous catheter, internal jugular vein, internal thoracic vein

## Abstract

Central venous catheter (CVC) placement is an essential component of critical care medicine. CVC malposition is a known complication of internal jugular vein (IJV) cannulation. However, catheterization of the internal thoracic vein (ITV) is much rarer. Only a handful of case reports have been documented, and guidelines for management are therefore lacking. Our case study describes this rarely occurring ITV cannulation along with the discussion of risk factors, warning signs of malpositioning, and subsequent management plans to optimize patient safety. Previous studies have used fluoroscopy and agitated saline flush tests to confirm that agents administered through an ITV-located catheter would reach the right atrium. Considering this, it would follow that a catheter in this site could theoretically be used for medication administration, especially in emergency settings. This hypothesis remains the most novel part of our case study and might prompt further exploration of management strategies in this particular situation.

## Introduction

Central venous catheter (CVC) placement is required to provide effective critical care, with common indications including volume resuscitation, hemodynamic monitoring, and administration of vasopressors, blood products, and parenteral nutrition. However, it is not without risks. Complications of CVC placement have been reported at rates as high as 15%. They commonly include, but are not limited to, pneumothorax, hemothorax, nerve injury, arteriovenous fistula formation, catheter fragmentation with subsequent embolization, and catheter malposition [1]. Cannulation of the internal jugular vein (IJV) is generally considered safer compared to the subclavian vein as it is readily accessible and rostral in location (therefore less likely to cause a pneumothorax) [2]. This case report describes the rare complication of accidental left internal thoracic vein (ITV) cannulation during an attempt to establish central access via the IJV. The first documented case of this phenomenon was reported in 1974 [2]. However, only a handful of similar case reports have since been documented, and guidelines for management are therefore lacking. This report discusses risk factors and warning signs of ITV cannulation as well as subsequent management to optimize patient safety.

## Case presentation

**Figure 1:**
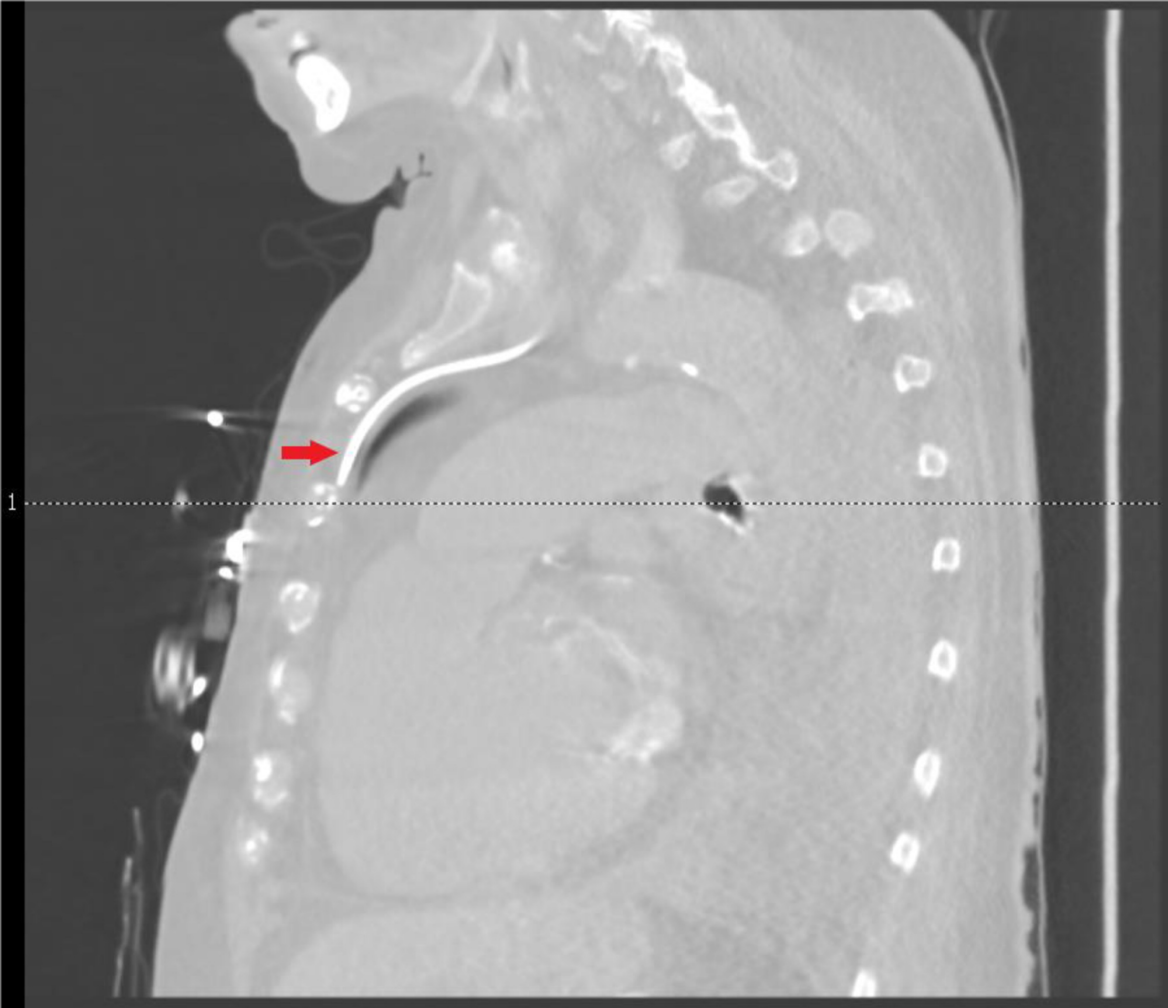
CT chest sagittal view of IJV CVC (red arrow) coursing along the anterior mediastinum, consistent with placement in the left ITV. IJV, internal jugular vein; CVC, central venous catheter; ITV, internal thoracic vein

**Figure 2:**
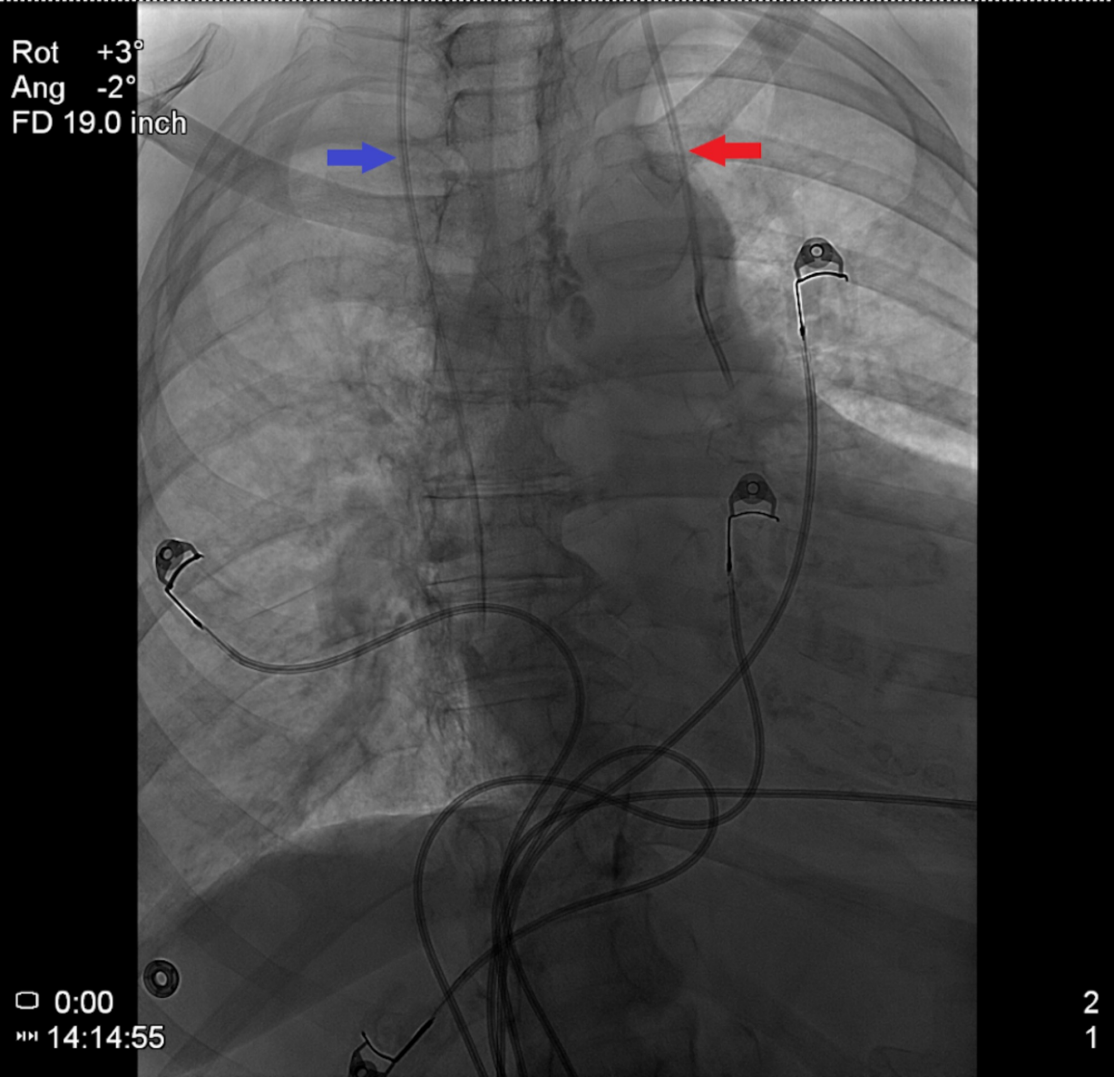
Chest radiograph with right-sided CVC (blue arrow) as well as the malpositioned left-sided catheter in the left IJV (red arrow), prior to removal of the latter. CVC, central venous catheter; IJV, internal jugular vein

A 76-year-old female in the ICU for septic shock required vasopressor support, which was initially administered via a right IJV CVC. However, new central venous access was required after the patient removed the aforementioned CVC during an episode of delirium. Left-sided IJV cannulation was achieved with ultrasound guidance. No resistance was encountered upon guidewire and subsequent catheter introduction to a depth of approximately 17 cm. Aspiration through the proximal port was without issue. However, initial aspiration attempts via the medial and distal ports failed. The catheter was advanced by approximately 2 cm with subsequent successful aspiration through all three ports. Chest X-ray (CXR) revealed the CVC following a vertical course along the left mediastinum, and transducer pressure measurement was consistent with venous placement. A CT scan of the chest confirmed cannulation of the left ITV (Figure 1). Interventional radiology (IR) was consulted to place a right-sided IJV catheter as the site had been previously cannulated. After the successful placement of the right-sided CVC, the left-sided catheter was removed without further complications (Figure 2).

 

## Discussion

The CVC malposition is a known complication of IJV cannulation, occurring at the estimated rate of 1%-2% [3-4]. However, ITV cannulation is a much rarer event while attempting access via the left IJV. ITV receives the anterior intercostal veins as well as some abdominal branches, eventually draining into the brachiocephalic vein behind the sternal end of the clavicle and first costal cartilage [3-4]. Therefore, it makes sense that proposed risk factors for left ITV cannulation include portal hypertension as well as the location of a patient’s left ITV directly opposite the left IJV orifice [1, 4]. In a patient with portal hypertension, blood flow from the portal system is diverted through collateral anastomoses, which in turn increases systemic circulation volume. This results in the dilation of small vessels in close proximity to these anastomoses, including the ITV. However, our patient did not have a history of portal hypertension, and more studies would be helpful in exploring other possible risk factors for ITV cannulation. Early signs of CVC misplacement include chest pain, guidewire resistance, and, as seen in our case, difficulty aspirating, particularly from the distal port(s) [5-6]. Although ultrasound is useful in confirming the intraluminal positioning of the guidewire, this case demonstrates that it is not sufficient to confirm catheter tip location [1]. CXR can only approximate catheter position, and prompt CT or CT angiography (CTA) should be strongly considered in these cases to rule out potentially disastrous complications [3]. If extravascular placement or placement in a large, incompressible artery or vein is suspected, discussion with IR and/or vascular surgery is recommended. Previous case reports have used fluoroscopy and agitated saline flush tests to confirm that agents administered through an ITV-located catheter would indeed reach the right atrium [1, 4]. Considering this, it would follow that a catheter in this site could theoretically be used for medication administration, especially in an emergency where time is of the essence. Indeed, one case report was documented using a catheter known to be located in the left ITV for four days without complications [6]. This hypothesis remains the most novel part of our case study and might prompt further exploration of management strategies in this situation.

## Conclusions

The CVC cannulation of the ITV is a very rare complication of attempted central venous access via the IJV. Due to the limited ability of CXR to approximate catheter position, prompt CT or CTA should be considered to rule out serious complications when CVC malposition is suspected. Vascular surgery and/or IR consultation may be needed. If confirmation of ITV cannulation is obtained with reasonable certainty, then the CVC should be removed as soon as it is practical. Further studies are needed to establish guidelines regarding further management of this event. 
